# Rhythmic Signaling of Ants and Butterflies With Varying Degrees of Myrmecophily

**DOI:** 10.1111/nyas.70223

**Published:** 2026-02-24

**Authors:** Chiara De Gregorio, Izabela Sondej, Stefano Previdi, Francesca Barbero, Luca Pietro Casacci

**Affiliations:** ^1^ Department of Psychology University of Warwick Coventry UK; ^2^ Department of Natural Forests Forest Research Institute Białowieża Poland; ^3^ Department of Life Sciences and Systems Biology University of Turin Torino Italy

**Keywords:** double meter, interspecific signaling, isochrony, rhythmic patterning

## Abstract

Myrmecophilous organisms have evolved in several arthropod lineages, developing specialized traits to communicate with ants. In butterflies, these include morphological, visual, chemical, behavioral, and acoustic adaptations. While acoustic communication has long been overlooked, recent studies show that vibrational signals mediate key aspects of caterpillar–ant interactions. Yet, no study has specifically investigated the rhythmic structure of such signals in myrmecophilous contexts, despite growing evidence that rhythm is a fundamental component of signal architecture across taxa. We examine the rhythmic properties of vibroacoustic signals from two ant and nine butterfly species differing in myrmecophily degree. We tested whether rhythmic features such as pulse train tempo, intertrain interval, and rhythmic patterns vary across taxa and reflect the strength of their ecological association with ants. Our results reveal that ants and highly myrmecophilous species share a complex rhythmic organization (isochrony and double meter), likely reflecting convergent adaptation to tight mutualistic interactions. Species with intermediate or no myrmecophily associations showed more variable or simplified rhythms. Temporal regularity and precision could possibly balance the need for signal recognizability with energetic constraints and avoidance of detection by unintended receivers. These findings highlight the role of temporal patterning in vibroacoustic communication, influencing signal efficiency and recognition in ant–butterfly interactions.

## Graphical abstract

Myrmecophilous butterflies evolved diverse adaptations to communicate with ants, including acoustic ones. We analyzed the rhythmic properties of vibroacoustic signals in two ant and nine butterfly species differing in myrmecophily. Highly myrmecophilous species and ants shared complex rhythmic structures (isochrony, double meter), suggesting convergent evolution for mutualistic coordination. Species with weaker associations showed simpler or irregular rhythms, highlighting rhythm's key role in signal efficiency and recognition.

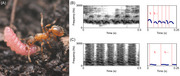

## 1 Introduction

Communication plays a central role in animal behavior, enabling the transfer of information between individuals through a variety of sensory channels. Among these, vibroacoustic communication is a specific form of signaling that involves the production and detection of mechanical vibrations, typically transmitted through air or various substrates such as soil, leaves, or plant stems [1]. Although across invertebrates chemical communication represents the dominant modality of communication, vibroacoustic signaling is also widespread, especially among several arthropod taxa, including web‐building spiders, crickets and katydids, leafhoppers, and planthoppers, where it serves key functions such as prey detection, mate attraction, and territorial interactions [2, 3].

In ant colonies, vibroacoustic signals are involved in a wide range of vital social functions, such as coordinating alarm and defense responses, marking territory, attracting mates, recruiting nestmates, initiating rescue behaviors, and conveying information related to caste or social hierarchy [4, 5, 6, 7]. Despite their vital role in the organization of ant colony life, vibroacoustic signals can also become a point of vulnerability. Several myrmecophilous organisms, that is, species that live in close association with ants, have evolved, besides the ability to imitate chemical signals, the capacity to mimic ant vibrational signals to manipulate their behavior, gaining access to food, protection, or integration within the colony [8, 9, 10, 11, 12].

This phenomenon has been particularly well studied in lycaenid butterflies (Lepidoptera: Lycaenidae), a family in which over 75% of species establish facultative or obligate associations with ants [13, 14, 15, 16]. Since the pioneering work of DeVries [17], it has become clear that both caterpillars and pupae of several lycaenid species are capable of emitting substrate‐borne vibrational signals, often functioning as acoustic mimicry to manipulate the behavior of their host ants [8]. By producing vibrational signals, lycaenid caterpillars could recruit, retain, or modulate the tending workers, often eliciting trophallaxis or protective behaviors [7]. On the other hand, ants respond to these signals with increased antennation, exploration, or context‐dependent actions such as digging [9, 10]. In particular, studies on obligate myrmecophilous *Phengaris* (= *Maculinea*) butterflies have shown that their caterpillars and pupae produce signals closely resembling those of ant queens, which elicit strong guarding and rescue responses by *Myrmica* workers [9, 10]. The acoustic properties of these signals have been shown to vary across species with different degrees of myrmecophily, suggesting an evolutionary link between signal structure and the level of host integration [7, 18].

However, the role of the temporal and rhythmic structure of vibroacoustic signals in effectively mediating communication between ants and their symbionts remains largely unexplored. In particular, the extent to which rhythm influences signal transmission and recognition in these systems is still unknown. However, studies on rhythmic structure may provide hints about its potential functional roles. Rhythm may play a key role not only in enhancing signal detectability and discrimination [19, 20] but also in conveying information through the temporal regularity of the signal itself [21]. Investigating rhythmic structure can thus provide important insights into how vibratory signals are produced, perceived, and function in interspecific and intraspecific communication.

This study aimed to investigate the rhythmic properties of insects' vibroacoustic signals to uncover the role of temporal patterning in within and between‐species communication. In particular, our first aim was to test whether the rhythm of vibroacoustic signals differs between the species examined, to understand the degree of species‐specificity, as this may reflect the extent to which signal properties are evolutionarily tuned to particular ecological or social interactions, such as species recognition. A high degree of species‐specificity suggests finely adapted communication strategies, while more conserved patterns indicate general constraints or shared evolutionary pressures.

We also investigated the degree of rhythmic similarity between the vibroacoustic signals produced by ants and those emitted by butterfly caterpillars, with a specific focus on whether the degree of myrmecophily could predict variation in temporal and rhythmic features. Given the importance of rhythmic patterning in animal communication, we tested the hypothesis that higher levels of myrmecophily would be associated with greater rhythmic similarity to ants. To address this, we examined four main aspects: (1) Are caterpillar trains faster or slower than ant signals? We predicted that species with higher myrmecophily would match ant tempo more closely. (2) Do caterpillars differ from ants in the timing of pauses between trains, and is this linked to myrmecophily? Higher myrmecophily species were expected to show intervals more similar to ants. (3) Do caterpillar signals exhibit the same rhythmic structures as ants (e.g., isochrony or double meter)? We hypothesized that species closely associated with ants would display more ant‐like rhythmic patterns. (4) How consistent are the trains in maintaining rhythmic structure? We predicted that species with stronger ant associations would show higher regularity. Together, these analyses enable us to investigate whether temporal and rhythmic convergence in vibroacoustic signaling may reflect adaptive adjustments linked to the strength of ant–butterfly associations, potentially shedding light on the evolutionary pressures shaping interspecific communication.

## 2 Methods

### 2.1 Data Collection

**FIGURE 1:**
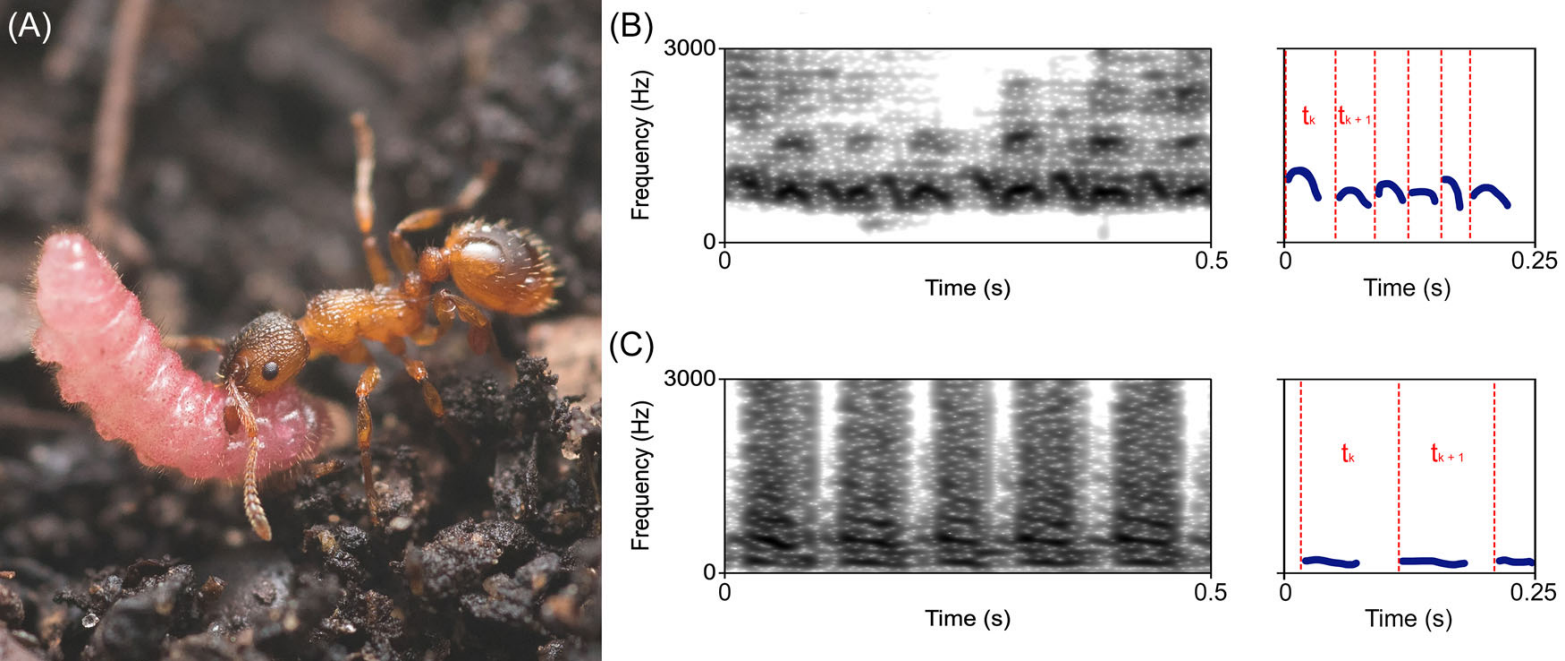
(A) *Phengaris alcon* caterpillar carried by a *Myrmica* worker. (B) Spectrogram of a *Myrmica* vibroacoustic signal with schematic *t*_k_ calculation. (C) Spectrogram of a *P. alcon* signal with corresponding *t*_k_ calculation.

Sounds produced by caterpillars (Figures 1 and S1) of nine lycaenid butterfly species, that is, *Lycaena dispar*, *L. phlaeas*, *Cupido argiades*, *Polyommatus icarus*, *P. bellargus*, *P. coridon*, *Scolitantides orion*, *Plebejus argus*, and *Phengaris* (= Maculinea) alcon, as well as two ant species of the genera *Myrmica* and *Tetramorium*, collected from various locations in Northern Italy, were recorded and compared. These genera are also widespread in European grasslands and often co‐occur with lycaenid caterpillars, making them ecologically relevant reference models for examining broader patterns of temporal convergence. All butterfly species exhibiting myrmecophily in this study belong to the subfamily Polyommatinae, whereas *L. dispar* and *L. phlaeas* are members of the subfamily Lycaeninae. Data on the butterfly and ant species used in this study were originally collected by Riva et al. [18]. Caterpillars and ant colonies were collected from various locations in Northern Italy between May 2012 and April 2014. Whenever possible, fully developed butterfly caterpillars were collected; otherwise, plants on which eggs had been laid were gathered to rear caterpillars in the laboratory. Specimens and their host plants were kept in transparent boxes (30 × 20 × 20 cm^3^) covered with mesh at temperatures of 18°C during the night and 25°C during the day, under a 14‐h light/10‐h dark cycle. After experiments, adults were released back into their original collection sites. Concurrently, ant nests were also collected in the field and subsequently maintained in plastic boxes (28 × 15 × 10 cm^3^) under controlled laboratory conditions and reared on a diet of sugar and *Drosophila* larvae. Selection of lycaenid species was based on their varying degrees of interaction with ants, classified following Fiedler [13] slightly modified by Schönrogge et al. [7] 0: no association with ants (absence of myrmecophily); 1: unstable associations with ants, occurring only occasionally (weakly myrmecophilous); 2: variable caterpillar attendance by ants (moderately myrmecophilous); 2.5: between moderately and constantly myrmecophilous; 3: most or all mature caterpillars consistently associated with ants (constantly myrmecophilous); 3.5 between constantly and obligate myrmecophilous; 4: caterpillars entirely dependent on ants as parasites (obligate myrmecophilous). For the statistical analyses, species of butterflies were grouped into four categories of myrmecophily based on previously assigned scores:

‐ No myrmecophily (“NO”)—*L. dispar*, *L. phlaeas* (score = 0)
‐ Low myrmecophily (“Low”)—*C. argiades* (score = 2), *P. icarus* (score = 2.5)
‐ Medium myrmecophily (“Medium”)—*P. bellargus*, *P. coridon*, *S. orion* (score = 3)
‐ High myrmecophily (“High”)—*P. argus* (score = 3.5), *P. alcon* (score = 4)

Sound recordings of late‐instar caterpillars and ants were obtained using a custom‐made device designed to minimize stress and disturbance. Samples were not stimulated/threatened with any tools to obtain the recordings. The recording setup included a compact chamber (12.5 × 8 × 2 cm^3^) equipped with a highly sensitive moving‐coil miniature microphone (sensitivity: 2.5 mV/Pa at 1 kHz), with a second identical microphone recording ambient noise in antiphase to reduce interference. Signals from both microphones were combined through a mixer and amplifier system covering a frequency range of 20 Hz to 20 kHz with a total gain of approximately 83 dB. The entire setup was powered by a 12 V gel cell battery and housed inside an anechoic chamber to minimize background noise. Individual caterpillars and ants were placed directly on the microphone surface and recorded at room temperature (23°C–25°C) for 20‐min intervals, beginning 5 min after placement. For full methodological details, see Riva et al. [18].

### 2.2 Data Preprocessing

Acoustic recordings were carefully inspected for quality, saved in WAV format (16‐bit resolution), and analyzed for temporal and spectral properties using Praat software. We obtained a total of 56 recordings, each one from a different subject: 11 for *Myrmica*, 12 for *Tetramorium*, five for *P. alcon*, five for *C. argiades*, three for *P. argus*, five for *P. bellargus*, one for *P. coridon*, two for *L. dispar*, five for *P. icarus*, four for *S. orion*, three for *L. phlaeas*.

Using the TextGrid tool in Praat (version 6.2.05), through visual inspections of spectrograms (Figure S1), we annotated the onsets and offsets of all pulses (i.e., call fundamental unit) emitted in each recording and measured the starting point and duration of each interval [22]. Moreover, since pulses occur in temporally structured sequences, we defined “pulse trains” as groups of regularly spaced pulses forming the basic units of caterpillar calls. Each pulse generally exhibits a harmonic structure composed of at least three frequency components, with the first corresponding to the fundamental frequency [18]

Using the software RStudio to evaluate the rhythmic structure of the vibroacoustic signals, we calculated the duration between the onsets of each couple of adjacent pulses (interonset interval or IOI; hereafter *t*_k_). We obtained 5581 *t*_k_ values for *Tetramorium*, 8754 for *Myrmica*, 5324 for *P. alcon*, 1507 for *P. argus*, 6643 for *P. bellargus*, 2392 for *P. coridon*, 7239 for *S. orion*, 5817 for *P. icarus*, 5897 for *C. argiades*, 417 for *L. dispar*, and 869 for *L. phlaeas*. Then, we calculated rhythmic ratios (*r*_k_) by dividing each interval *t*_k_ for itself plus the following one: *r*_k_ = *t*_k_/(*t*_k_ + *t*_k + 1_; see Refs. [23, 24, 25]). We obtained 5070 *r*_k_ for *Tetramorium*, 8474 for *Myrmica*, 5263 for *P. alcon*, 141 for *P. argus*, 6500 for *P. bellargus*, 2384 for *P. coridon*, 6532 for *S. orion*, 5663 for *P. icarus*, 5480 for *C. argiades*, 321 for *L. dispar*, 643 for *L. phlaeas*.

To evaluate the occurrence of small‐integer ratios, we tested whether the number of data points falling within predefined ratio boundaries is significantly greater than expected under a null distribution. In particular, we were interested in investigating the presence of the two simplest small‐integer ratios: isochrony (1:1), when two consecutive temporal units have the same duration (e.g., the ticking of a metronome), and double meter (1:2), which refers to rhythmic patterns in which one event is followed by another that is approximately twice as long or short in duration. To do so, we divided the ratio distribution into on‐integer and off‐integer ratio ranges, and we centered the on‐integer range around 0.5 (or 1:1), 0.33 (or 1:2), and 0.66 (or 2:1). In accordance with Ref. [23], a 1:1 on‐integer ratio is considered when ratio values fall within the values between 0.444 and 0.555, and an off‐integer ratio when it is between 0.4–0.444 and 0.555–0.6. The range of the 1:2 on‐integer ratio is 0.308–0.364, while the off‐integer ratio is within 0.286–0.308 and 0.364–0.4. Finally, for the 2:1 ratio, the on‐integer ratio is within 0.636–0.692, while the respective off‐integer ratio is within 0.6–0.636 and 0.692–0.714. We then tallied all the ratios occurring within the respective on‐integer and off‐integer ratio intervals for each group of pulses (trains) contained in each file.

### 2.3 Statistical Analyses

We performed all statistical analyses below using RStudio. We first investigated differences in tempo and interval duration at the species level, and then at the myrmecophily degree level. We ran a total of 12 models. To test for the significance of our full models, we compared them against null models containing only the random factors [26] with a likelihood ratio test (ANOVA with argument “chi‐sq” [27]).

#### 2.3.1 Rhythmic Differences Between Species: Tempo of Trains and Intervals

To test whether the tempo of trains and intervals between trains differed across species, we used linear mixed models (LMMs; *lme4* package [28]) with species identity as a fixed factor and file ID as a random factor, followed by post hoc pairwise comparisons among species (*emmeans* package [29]). The response variables were log‐transformed, and in both cases the full model differed significantly from the null (tempo of trains: χ^2^ = 34.42, df = 10, *p* < 0.001; intervals between trains: χ^2^ = 81.54, df = 10, *p* < 0.001).

#### 2.3.2 Rhythmic Differences Based on the Myrmecophily Degree

We ran a total of two LMMs and eight generalized linear mixed models (GLMMs), using a null versus full approach: to test for the significance of our full models, we compared each model with the same one containing random factors only (ANOVA with chi‐sq argument [27]). If null and full significantly differed, then the fixed factors affected the distribution of the response variable, and we therefore applied post hoc tests (*emmeans* package [29]) to perform all pairwise comparisons for the levels of the fixed factors. Before fitting the models, we log‐transformed the duration values for the LMMs and used Poisson as the distribution for the GLMMs (except for two cases—the one focusing on butterflies with a low degree of myrmecophily was fitted as negative binomial after checking the overdispersion parameter; the one testing regularity rate was fitted with a beta distribution).

#### 2.3.3 Tempo

To test whether the tempo of pulses (in trains) emitted by caterpillars differed from the ones emitted by ants, we used a LMM (*lme4* package, [28]). We used the log‐transformed duration of *t*_k_ as the response variable, with the level of myrmecophily (No, Low, Medium, High) as a fixed factor, and the ID of the file from which *t*_k_ were extracted as a random factor. The full model significantly differed from the null one (Null vs. Full—χ^2^ = 18.56499, df = 4, *p* < 0.001). We then ran a post hoc test to perform all pairwise comparisons for all levels of the variable “myrmecophily degree” (*emmeans* package [29]).

#### 2.3.4 Interval Duration

To test whether the duration between different trains of pulses emitted by caterpillars differed from the ones emitted by ants, we used a LMM (*lme4* package [28]). We used as a response variable the log‐transformed duration of intervals, as a fixed factor the level of myrmecophily (No, Low, Medium, High), and as a random factor the ID of the file from which intervals were extracted. The full model significantly differed from the null one (Null vs. Full—χ^2^ = 12.91156, df = 4, *p* = 0.012). We then ran a post hoc test to perform all pairwise comparisons for all levels of the variable “myrmecophily degree” (*emmeans* package [29]).

#### 2.3.5 Rhythmic Organization

We used five GLMMs (*glmmTMB* package [30]), one for each level of category (ants and the four levels of myrmecophyly: No, Low, Medium, High) to investigate if caterpillar trains are temporally organized as ants’ ones. In particular, we tested whether their rhythmic organization would match an isochronous peak (1:1 ratio) or double meter (1:2 and 2:1 rhythmic ratios). For each model, our response variable was the number of *r*_k_ for each level, as a fixed factor *r*_k_ bin type (factor levels: OFF1:1, ON1:1, OFF1:2, ON1:2, OFF2:1, ON2:1). For all models, we used the file ID as a random factor. We accounted for the presence of zeros in the dataset by specifying ziformula = 1. Additionally, we included an offset term to adjust the *r*_k_ counts relative to the width of each bin in the probability density function, thus controlling for differences in bin size. The comparison with the null model was significant for all models (Ants: χ^2^ = 10,783.05, df = 5, *p* < 0.001; High degree: χ^2^ = 2215.421, df = 5, *p* < 0.001; Medium degree: χ^2^ = 7419.142, df = 5, *p* < 0.001; Low degree: χ^2^ = 4911.878, df = 5, *p* < 0.001; No myrmecophily: χ^2^ = 1666.756, df = 5, *p* < 0.001). We then ran a post hoc test (*emmeans* package [29]) for multiple comparisons between observations falling into each bin type.

#### 2.3.6 Regularity Rate

To investigate possible differences in terms of regularity rate between species with high, low, medium, and no myrmecophily and ants, we calculated the regularity rate for the rhythmic peak as the ratio between the count of on‐integer observations and on‐plus off‐integer observations of the individual contribution. This metric captures the temporal regularity of signal production, indicating how closely the trains approximate a consistent rhythmic pattern (e.g., 1:1, isochrony; 1:2 and 2:1, double meter). We ran three GLMMs (*glmmTMB* package [30]), one for each rhythmic category investigated (1:1, 1:2, 2:1). For each model, we used the rate of regularity as the response variable, as a fixed factor the level of myrmecophily (No, Low, Medium, High), and as a random factor the ID of the file from which rate values were calculated. For the model investigating the regularity of a 1:1 ratio, the full model differed significantly from the null one (Null vs. Full—χ^2^ = 87.656916, df = 4, *p* < 0.001). Conversely, for the 1:2 and 2:1 ratios, the full models did not significantly differ from the null ones (1:2, χ^2^ = 2.431607, df = 1, *p* = 0.118; 2:1, χ^2^ = 0.7425669, df = 1, *p* = 0.388).

## 3 Results

### 3.1 Rhythmic Differences Between Species

#### 3.1.1 Tempo

**FIGURE 2:**
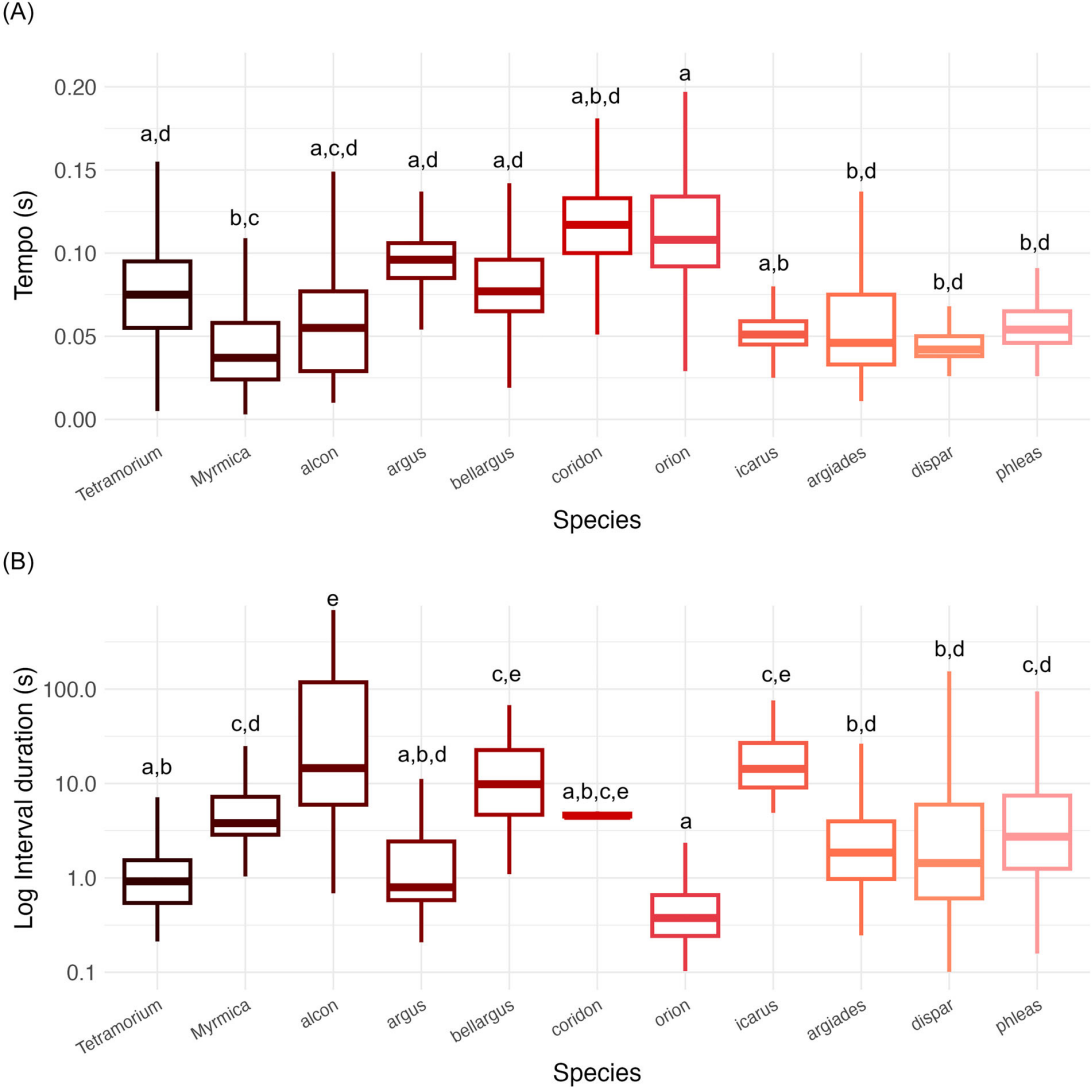
Temporal features of vibroacoustic communication in ants and butterflies. (A) Boxplot showing *t*_k_ value per species. (B) Boxplot showing the interval duration (log) between trains of pulses per species. Letters above each column represent the outcome of post hoc pairwise tests (Tables S1 and S2). Species sharing the same letter do not differ significantly (*p* > 0.05).

Rhythmic differences in the tempo of pulses (in trains) were observed between species. In particular, we found (i) Differences between ant species with *Myrmica* having faster trains than *Tetramorium* (*Myrmica* vs. *Tetramorium*, *p* = 0.031, Figure 2A). (ii) Differences among caterpillar species, with *C. argiades* trains faster than *S. orion*’s ones (*argiades* vs. *orion*, *p* = 0.025), as well as *L. dispar*’s, and *L. phlaeas*’ (*dispar* vs. *orion*, *p* = 0.032; *orion* vs. *phlaeas*, *p* = 0.024, Figure 2A). Moreover, *P. argus* vibroacoustic signals showed only a tendency toward being slower than *L. phlaeas*’ ones (*argus* vs. *phlaeas*, *p* = 0.068). (iii) Differences between ants and caterpillars, in particular *Myrmica* ants produced trains with a faster tempo than *P. bellargus* caterpillars (*bellargus* vs. *Myrmica*, *p* = 0.015; Figure 2A), *P. argus* (*argus* vs. *Myrmica*, *p* < 0.001), and *S. orion* (*Myrmica* vs. *orion*, *p* < 0.001). All the other comparisons were not significant (Table S1).

#### 3.1.2 Interval Duration

Interval duration between trains differed across species (Table S2). Notably, the two ant species differed in the duration of intervals between trains (*Tetramorium* vs. *Myrmica*, *p* = 0.002, Figure 2B), and *Tetramorium* ants had shorter intervals than *P. alcon* (*Tetramorium* vs. *alcon*, *p* < 0.001). Focusing on the differences between interval durations within butterfly species, we found that *P. alcon* intervals were longer than *C. argiades* (*alcon* vs. *argiades*, *p* < 0.001), *P. argus* (*alcon* vs. *argus*, *p* < 0.001), *L. dispar* (*alcon* vs. *dispar*, *p* = 0.005), *S. orion* (*alcon* vs. *orion*, *p* < 0.001, Figure 2B), and *L. phlaeas* (*alcon* vs. *phlaeas*, *p* = 0.027). *C. argiades* intervals were longer than *S. orion*s ones (*argiades* vs. *orion*, *p* = 0.003), while shorter than *P. icarus* (*argiades* vs. *icarus*, *p* = 0.003). We also found a tendency for intervals in *C. argiades* being shorter than *P. bellargus* intervals (*argiades* vs. *bellargus*, *p* = 0.055). *P. argus* caterpillars had shorter intervals than *P. icarus* caterpillars (*argus* vs. *icarus*, *p* = 0.005), while they showed only a tendency to be shorter than *P. bellargus* (*argus* vs. *bellargus*, *p* = 0.056). Moreover, on top of emitting shorter intervals than *P. alcon* and *C. argiades*—as previously reported—*S. orion* caterpillars emitted intervals between trains that were also shorter than *P. bellargus* (*bellargus* vs. *orion*, *p* < 0.001, Figure 2B), *P. icarus* (*icarus* vs. *orion*, *p* < 0.001, Figure 2B), and *L. phlaeas* (*orion* vs. *phlaeas*, *p* < 0.001), and a tendency to be shorter than *L. dispar* ones (*dispar* vs. *orion*, *p* = 0.052). Finally, *L. dispar* caterpillars had intervals between trains that were shorter than *P. icarus* ones (*dispar* vs. *icarus*, *p* = 0.048).

Finally, we also found that the duration of intervals differed between caterpillars and ants. In particular, *Tetramorium* had shorter intervals than *P. bellargus* (*Tetramorium* vs. *bellargus*, *p* < 0.001), *P. icarus* (*Tetramorium* vs. *icarus*, *p* < 0.001, Figure 2B), and *L. phlaeas* caterpillars (*Tetramorium* vs. *phlaeas*, *p* = 0.027). *Myrmica* ants differed from *P. alcon* caterpillars too (*Myrmica* vs. *alcon*, *p* = 0.004), but compared to *Tetramorium*, we found only a tendency toward having significantly shorter intervals than *P. icarus* caterpillars (*Myrmica* vs. *icarus*, *p* = 0.069), and they also had shorter intervals between trains than *S. orion* caterpillars (*Myrmica* vs. *orion*, *p* < 0.001, Figure 2B). No other comparisons were significant (see Table S2).

### 3.2 Rhythmic Differences Based on the Degree of Myrmecophily

#### 3.2.1 Tempo

**FIGURE 3:**
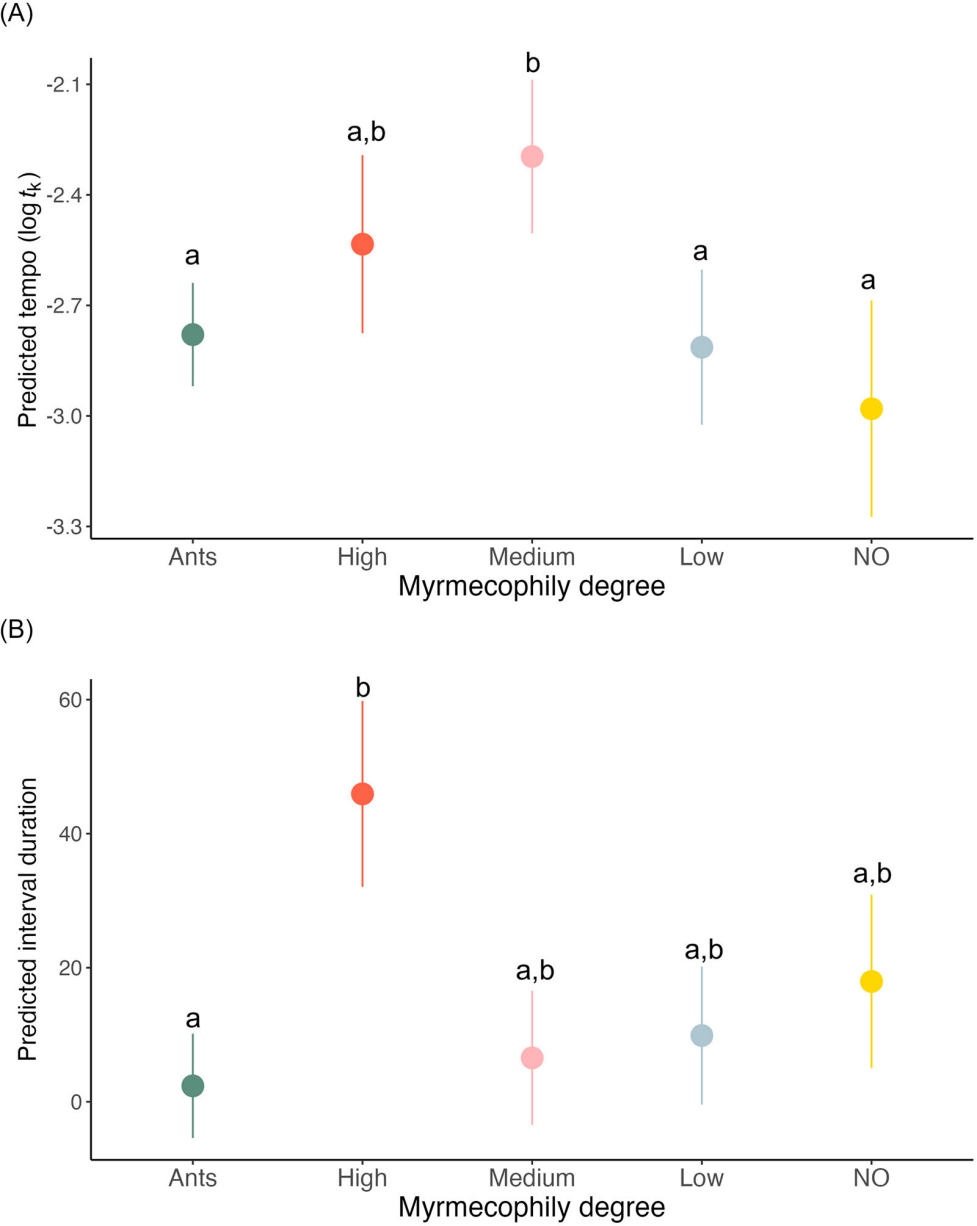
Predicted values of temporal features across species grouped by myrmecophily degree. (A) Predicted values of the tempo of pulses within a train. (B) Predicted values of intervals between trains. Points represent model estimates with 95% confidence intervals. Predicted values are derived from the fixed effects of the full model. Species sharing the same letter do not differ significantly (*p* > 0.05).

Butterfly species with a medium degree of myrmecophily exhibited a different tempo compared to ants and other caterpillars. In particular, they had a slower tempo of trains than ants (*Medium* vs. *ants*, *p* = 0.002, Figure 3A and Table 1), a slower tempo than nonmyrmecophilous species (*NO* vs. *medium*, *p* = 0.002, Figure 3A and Table 1), and a slower tempo than low‐degree myrmecophily caterpillars (*Low* vs. *medium*, *p* = 0.006, Figure 3A and Table 1).

**TABLE 1:** Post hoc comparisons of the LMM testing for the effect of the degree of myrmecophily on the tempo of pulses.

Contrast	Estimate	SE	*Z* ratio	*p* value
NO—Low	−0.167	0.184	−0.907	0.894
NO—Medium	−0.685	0.184	−3.731	**0.002**
NO—High	−0.447	0.194	−2.304	0.143
NO—Ants	−0.201	0.166	−1.214	0.743
Low—Medium	−0.518	0.151	−3.430	**0.006**
Low—High	−0.280	0.163	−1.712	0.426
Low—Ants	−0.034	0.129	−0.267	0.999
Medium—High	0.238	0.163	1.462	0.588
Medium—Ants	0.483	0.128	3.776	**0.002**
High—Ants	0.245	0.143	1.722	0.420

*Note*: *p*‐values in bold indicate statistical significance (*p* < 0.05).

#### 3.2.2 Interval Duration

Caterpillar characterized by a high level of myrmecophily showed longer intervals between trains than ants (*High* vs. *Ants*, *p* = 0.020, Figure 3B), while no other differences were detected (Table S3).

#### 3.2.3 Rhythmic Organization

We compared the number of *r*_k_ (ratios) falling in the on‐integer bin (e.g., 1:1 on; ratio close to 0.5 = matching isochrony) versus the off‐integer bin (e.g., 1:1 off; ratio around 0.6 = not matching isochrony) for all three rhythmic ratios considered. For each group, the five models showed significant differences in *r*_k_ count across *r*_k_ bin types for the 1:1 ratio (Table 2). The vibroacoustic signal of all species exhibited a prominent isochronous component (*p* < 0.001, Table 2 and Figure S2). Ants and highly myrmecophilous caterpillars were the only groups that showed two additional rhythmic categories, corresponding to double meter (1:2, 2:1; *p* < 0.001 in all cases, Table 2).

**TABLE 2:** Post hoc comparisons of the five GLMMs testing for the effect of *r*_k_ bin type (off 1:1, on 1:1, off 1:2, on 1:2, off 2:1, on 2:1) on the *r*_k_ count in the five groups considered (Ants, High, Medium, Low, and NO).

Type	Contrast	Estimate	SE	*Z* ratio	*p* value
**Ants**	1:1 off–1:1 on	−0.537	0.025	−21.572	**< 0.001**
	1:2 off–1:2 on	−0.331	0.045	−7.316	**< 0.001**
	2:1 off–2:1 on	−0.426	0.046	−9.201	**< 0.001**
**High**	1:1 off–1:1 on	−1.010	0.0366	−28.328	**< 0.001**
	1:2 off–1:2 on	−0.381	0.072	−5.259	**< 0.001**
	2:1 off–2:1 on	−0.535	0.078	−6.854	**< 0.001**
**Medium**	1:1 off–1:1 on	−1.400	0.023	−61.153	**< 0.001**
	1:2 off–1:2 on	−0.080	0.067	−1.193	0.233
	2:1 off–2:1 on	−0.111	0.068	−1.617	0.106
**Low**	1:1 off–1:1 on	−1.810	0.070	−25.759	**< 0.001**
	1:2 off–1:2 on	−0.235	0.131	−1.801	0.072
	2:1 off–2:1 on	−0.217	0.137	−1.585	0.113
**No**	1:1 off–1:1 on	−1.530	0.091	−16.847	**< 0.001**
	1:2 off–1:2 on	0.372	0.358	1.038	0.300
	2:1 off–2:1 on	−0.190	0.338	−0.560	0.575

#### 3.2.4 Regularity Rate

**FIGURE 4:**
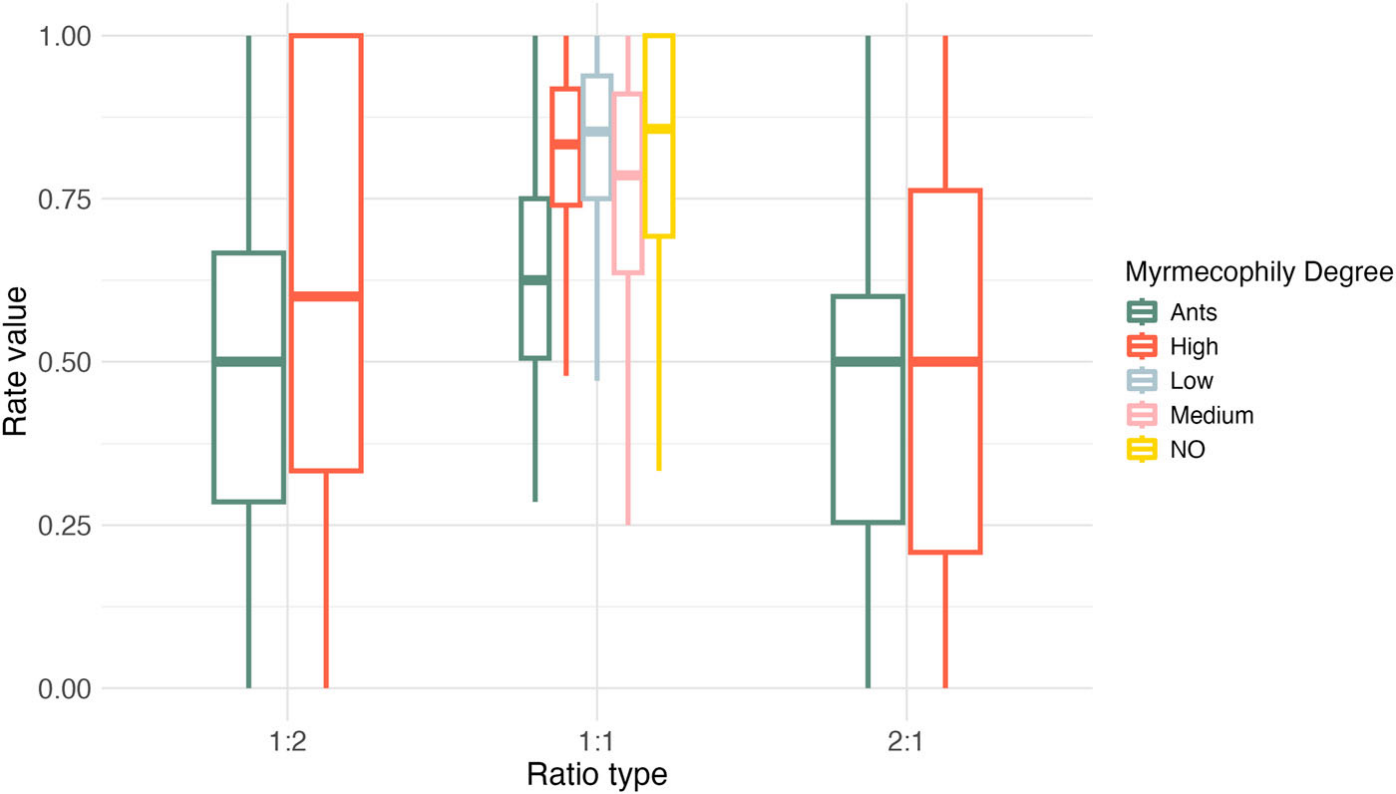
Boxplot showing the regularity rate for the studied groups considered for the three rhythmic ratios. For ratios 1:2 and 2:1, only two groups are shown because they are the only ones matching the rhythmic category. Number of files (each from a different individual) and *r*_k_ analyzed per group: Ants—23 recordings, 13,544 *r*_k_; High—eight recordings, 5404 *r*_k_; Medium—10 recordings, 15,416 *r*_k_; Low—10 recordings, 11,143 *r*_k_. No—five recordings, 964 *r*_k_.

Rhythm regularity varied with the degree of myrmecophily, with ants being less regular than all other groups (*p* < 0.001 for all contrasts; Table 3 and Figure 4). In turn, caterpillars with a medium degree of myrmecophily were less regular than the ones with low (*p* = 0.043) or no myrmecophily (*p* < 0.001). For the other two rhythmic ratios (1:2 and 2:1), since the full models did not significantly differ from the null ones, we can conclude that the level of myrmecophily does not explain the observed variation in these rhythmic ratios.

**TABLE 3:** Post hoc comparisons of the GLMM testing regularity rate for the 1:1 rhythmic ratio (isochrony).

Contrast	Estimate	SE	*Z* ratio	*p* value
NO—Low	0.405	0.189	2.140	0.203
NO—Medium	0.852	0.180	4.744	**< 0.001**
NO—High	0.408	0.261	1.561	0.522
NO—Ants	1.625	0.189	8.579	**< 0.001**
Low—Medium	0.448	0.161	2.783	**0.043**
Low—High	0.003	0.249	0.014	1.000
Low—Ants	1.221	0.172	7.096	**< 0.001**
Medium—High	−0.444	0.242	−1.838	0.352
Medium—Ants	0.773	0.160	4.835	**< 0.001**
High—Ants	1.217	0.249	4.888	**< 0.001**

## 4 Discussion

Our study revealed distinct rhythmic differences among the species investigated. In particular, the two ant species differed from each other, and both also showed rhythmic distinctions compared to some of the butterfly caterpillars. However, the temporal structures varied among species in a nonuniform way, with some species exhibiting unique signaling tempos or intervals, while others showed more similar temporal patterns. Such variation likely reflects species‐specific ecological or communicative adaptations but also indicates shared features in rhythmic organization across the group. Several studies have highlighted the species‐specific nature of vibrational signaling in insects. For example, Riva et al. [18] documented distinct vibroacoustic profiles in lycaenid caterpillars interacting with ants, showing that even closely related species exhibit differences in temporal and spectral signal parameters. Notably, part of the species and vibroacoustic recordings analyzed in the present study derive from the same dataset, allowing for a direct exploration of rhythmic features across previously characterized taxa. In a related context, Ferreira et al. [31] demonstrated that stridulation patterns can even be used to reveal cryptic speciation among sympatric neotropical ants, suggesting that acoustic signatures may encode fine‐grained species‐level distinctions that are not apparent from morphology alone. Taken together, these observations support the idea that the rhythmic profiles described in our study might offer complementary insights into subtle interspecific differences, particularly among species with convergent ecological interactions. Among the factors potentially shaping these rhythmic divergences, the degree of myrmecophily emerges as a particularly relevant axis of variation, influencing both the structure and regularity of vibrational signaling.

### 4.1 Isochrony as a Shared Temporal Structure, Double Meter Only in Ants and Highly Myrmecophilous Butterfly Species

Across all levels of myrmecophily examined in this study, the temporal structure of signaling behavior was consistently organized according to an isochronous pattern. However, the precision of this rhythmic pattern varied among species. This shared rhythmic organization suggests that isochrony may serve as a fundamental temporal template in caterpillar signaling, regardless of the degree of ant association. Isochrony has been documented across a wide range of animal taxa, from birds [23] and mammals [32], to various insect species, where it appears to enhance both signal transmission and perceptual processing. For example, Chievers et al. [33] showed that the orthopteran *Arachnoscelis arachnoides* produces discrete isochronous pulses when calling, a strategy that likely increases signal reach and reduces attenuation in dense forest environments. Similarly, in *Agnotecous lebinthus*, another orthopteran species, males emit sequences of isochronous trills during courtship, which are hypothesized to play a key role in female attraction and mate selection [34]. In the context of ant–caterpillar communication, maintaining isochrony may likewise increase the predictability and recognizability of the signal, thereby facilitating ant attention or approach, even when the mutualistic relationship is weak or facultative.

Moreover, our study shows an interesting case of a double meter rhythmic structure in insect vibroacoustic communication, a particularly complex form of rhythm that, to date, has been reported primarily in primates [32, 35, 36]. To date, the debate on the perceptual and functional significance of these rhythms remains open. Here, we aim to show that the temporal intervals in these signals tend to cluster around a 1:2 ratio rather than occurring by chance. This observation offers a starting point for future research and suggests that certain temporal structures may be deeply conserved across taxa, even if their communicative or functional roles may differ substantially from those observed in vertebrates. In our dataset, double meter was restricted to ants and the highly myrmecophilous caterpillars, suggesting a potential link between rhythmic complexity and tight ecological integration. This finding suggests that elaborate rhythmic structures, such as double meter, may have evolved convergently across distant taxa, potentially under shared selective pressures favoring signal efficiency and communicative clarity. In insects, such rhythmic complexity could enhance information encoding and improve signal discrimination in the context of mutualistic interactions, particularly within the nest where close ant–caterpillar associations typically occur. In these enclosed environments, changes in substrate, tunnel architecture, and acoustic constraints may favor more structured rhythmic patterns compared to looser, facultative interactions that take place outside the nest. This interpretation aligns with previous evidence that acoustic similarity among myrmecophilous species may evolve in response to ecological interaction strength rather than phylogenetic proximity [7] and expands on earlier findings that ant‐associated caterpillars display species‐specific vibrational patterns shaped by selection [18].

### 4.2 Ants and Highly Myrmecophilous Caterpillars

Ants and the highly myrmecophilous caterpillars shared not only the same pulse train tempo but also a similar rhythmic organization, suggesting a potential convergence shaped by close interspecific interaction. However, the caterpillars tended to exhibit longer intervals between trains. Species of the genus *Phengaris* have been shown to closely mimic key vibroacoustic characteristics of their *Myrmica* ant hosts, including dominant frequency, pulse length, and pulse repetition rate [9, 37, 38]. This is consistent with our findings, in which *Myrmica* ants and *Phengaris alcon* caterpillars exhibited similar pulse emission rates and shared a comparable rhythmic structure, indicating a high degree of temporal coordination. Notably, both ants and highly myrmecophilous caterpillars showed the same complex rhythmic organization comprising three different rhythmic categories. However, a key difference emerged in the duration of the intervals between pulse trains, which were longer in *P. alcon*. A reduction in signaling frequency may reflect lower urgency or an adaptive strategy to minimize energetic costs while reducing the likelihood of being parasitized, particularly within the ant nest, where signaling could inadvertently attract unintended receivers. Indeed, caterpillars’ acoustic signals could function analogously to chemical cues [39], providing parasitoids with species‐specific information that enables them to detect and identify their hosts even if hidden inside the nest.

We also found that highly myrmecophilous species exhibited greater precision in their isochronous rhythmic pattern than ants. Experimental studies on insects such as crickets and katydids have demonstrated that increased temporal regularity facilitates signal recognition and helps receivers extract relevant information from complex acoustic environments [40]. Temporal patterning has also been shown to improve signal detectability by increasing the signal‐to‐noise ratio, especially under noisy environmental conditions [41]. Although these findings concern airborne acoustic signals, similar constraints may apply to substrate‐borne vibrational signals in the crowded, intricate, and noisy context of subterranean ant colonies. Therefore, the enhanced rhythmic precision observed in caterpillar signals may similarly enhance effective communication with their ant partners. Another aspect to consider is that the lower regularity of ant vibrations may reflect their multifunctional use across diverse behavioral contexts. In contrast, the higher regularity of caterpillar signals likely stems from their specialized role in maintaining interactions with attendant ants.

### 4.3 Medium Level of Myrmecophily

Species with an intermediate degree of myrmecophily appear to lack a strong, stable relationship with ants, and as a result, their vibroacoustic signals may be more easily ignored by potential partners. These caterpillars displayed a slower train tempo, which may help sustain signaling over time with reduced energetic cost. Interestingly, the intervals between trains did not differ from those observed in ants, suggesting that intermediate species may slow down their trains while maintaining relatively short pauses to remain detectable. *P. coridon*, a species exhibiting intermediate levels of myrmecophily, attracts ants primarily through the secretion of nutritional rewards [42], suggesting that the maintenance of ant attendance entails a significant energetic cost. Such investment may correspond to the observed tendency to slow down their signaling rhythm, potentially as a strategy to balance signal duration with energy preservation.

Unlike highly myrmecophilous species, these caterpillars employed only a simple 1:1 rhythm, making their signaling patterns less similar to those of ants, possibly reflecting their ability to establish non–species‐specific associations with multiple ant hosts. Yet they were more regular in maintaining an isochronous pattern than ants, though less precise than species with no or low levels of myrmecophily. This suggests that while their signals may be partially tuned for ant communication, they still reflect a trade‐off between signal efficiency and ecological or physiological constraints. Species with intermediate levels of myrmecophily are known to associate with a high number of ant species spanning several genera, rather than relying on a single, tightly coevolved host. This broader ecological spectrum may favor signals that are generically recognizable by ants across multiple taxa rather than finely tuned to a specific ant partner. Notably, *P. coridon* has been recorded in association with at least 19 ant species from nine genera, while *P. bellargus* and *P. orion* also show a similarly wide range of associations [14]. This diversity of potential hosts suggests that, for intermediate species, rhythmic regularity may enhance signal recognition across a taxonomically diverse set of ants. Rather than evolving precise acoustic mimicry for a single host, these caterpillars might rely on generalizable, energetically balanced signals that function effectively within variable ecological contexts and host assemblages. This interpretation finds further support in the differential resemblance of the two ant species analyzed: while *Myrmica* showed signal traits convergent with highly myrmecophilous caterpillars, *Tetramorium* appeared acoustically more distant. Given that species with intermediate myrmecophily may interact opportunistically with both genera [14], the acoustic disparity between these ants could contribute to the heterogeneous vibroacoustic features observed in medium‐level associates, depending on which host species is locally available or ecologically dominant.

These differences in signaling rhythm and structure might also be influenced by the physical properties of the substrate where communication occurs. Species with moderate myrmecophily typically interact with ants on plant surfaces, where vibrational signals are strongly attenuated and filtered. Under these conditions, increasing signal duration or maintaining a more regular rhythm may enhance detectability and improve the chances of eliciting a response [42, 43].

### 4.4 Low or No Myrmecophily

Species with low or no myrmecophily displayed remarkably similar temporal signaling features among them: they shared interval durations, train tempo, rhythmic category (isochrony), and overall regularity. This convergence suggests that temporal signal structure is less affected when the association with ants is weak or absent, and that rhythmic modulation becomes more pronounced only at medium or high levels of myrmecophily. Interestingly, despite their weak association with ants, these caterpillars showed interval durations comparable to those of ants, although their train tempo differed from that of species with a medium degree of myrmecophily. This pattern may indicate that both high and low/no myrmecophilous species have more predictable or stereotyped signaling structures, possibly reflecting a well‐established relationship, either because fine‐tuned communication is unnecessary (in the case of no myrmecophily) or because the interaction is already well‐established (in the case of high myrmecophily). Species at the intermediate level, in contrast, may be engaged in more unstable, or context‐dependent interactions, which require dynamic adjustment in signaling.

In low or nonmyrmecophilous lycaenids, the presence of rhythmic, structured vibroacoustic signals suggests functions beyond ant interactions. Because their predictable patterns differ from the irregular bursts typical of defensive displays against predators [44], these signals could likely mediate intraspecific interactions such as territorial disputes (e.g., *Drepana arcuata* [45]) or rival assessment (e.g., *Falcaria bilineata* [46]), paralleling vibrational communication observed in other caterpillar species.

## 5 Conclusions

This study investigated the role of vibroacoustic rhythm in ants and butterfly caterpillars, focusing on how temporal patterns relate to the presence and intensity of myrmecophilous associations. We found that both ants and caterpillars show isochronous signals, and that ants and highly myrmecophilous species share a more complex rhythm, combining isochrony with double meter. This complex rhythmic organization, previously described mainly in primates, is here interpreted as a temporal pattern rather than as evidence of perceptual or functional complexity. Its recurrence across such distant taxa may point to a deeply conserved temporal organization that can be later co‐opted for different communicative roles, as enhancing signal efficiency in mutualistic contexts. The ability of mutualists and social parasites to reproduce certain acoustic features of their host ants [9, 37] supports the idea that vibroacoustic signals convey recognition cues important for interspecific interactions. Our results extend this evidence by highlighting rhythm, its tempo, and structural complexity as a potentially informative dimension in ant‐associated communication. Schönrogge et al. [7] suggested that the strength of ant–myrmecophile interactions correlates with distinctive acoustic profiles, and that these similarities may be better predicted by ecological interaction strength than by phylogenetic relatedness. Our data support the idea that acoustic similarity reflects ecological interaction strength in highly and nonmyrmecophilous species, but not a strict continuum in temporal traits. Moderately myrmecophilous species show more variable patterns, suggesting that factors beyond myrmecophily (such as substrate, energy constraints, or interactions with multiple ant species) also shape signal design. Future research should investigate the functional significance of rhythmic organization in insects, including its effects on communication, mate choice, and species recognition, while comparative studies across taxa could clarify the evolutionary and ecological drivers of these complex patterns.

## Author Contributions

**Chiara De Gregorio**: conceptualization. **Chiara De Gregorio**: methodology. **Chiara De Gregorio, Izabela Sondej, Stefano Previdi, Luca Pietro Casacci, and Francesca Barbero**: Investigation. **Luca Pietro Casacci and Francesca Barbero**: Supervision. **Chiara De Gregorio**: writing – original draft. **Chiara De Gregorio, Luca Pietro Casacci, Francesca Barbero, and Izabela Sondej**: writing – review and editing. **Chiara De Gregorio**: Visualization. All the authors contributed to the article and approved the submitted version.

## Conflicts of Interest

The authors declare no conflicts of interest.

## Supporting information




**Supporting Information Tables**: nyas70223‐sup‐0001‐Tables.docx

**Supporting Information Figures**: nyas70223‐sup‐0002‐Figures.docx

## Data Availability

The data that support the findings of this study are available from the corresponding authors upon reasonable request.

## References

[1] R. Dunlop , W. L. Gannon , M. Kiley‐Worthington , P. S. M. Hill , A. Wessel , and J. A. Thomas , “Vibrational and Acoustic Communication in Animals,” in Exploring Animal Behavior Through Sound, Volume 1: Methods, ed. C. Erbe and J. A. Thomas (Springer International Publishing, 2022), 389–417, 10.1007/978-3-030-97540-1_11.

[2] A. Wessel , S. Ehlers , K. W. McCravy , and J. A. Thomas , “Insect Bioacoustics and Biotremology,” in Exploring Animal Behavior Through Sound, Vol. 2, ed. Erbe, C. and Thomas, J. A. (Springer, 2025), 10.1007/978-3-031-83460-8_2.

[3] T. A. Mooney , L. Roberts , K. W. McCravy , and J. A. Thomas , “Invertebrates Other Than Insects,” in Exploring Animal Behavior Through Sound, Vol. 2, ed. Erbe, C. and Thomas, J. A. (Springer, 2025), 10.1007/978-3-031-83460-8_1.

[4] B. Hölldobler and E. O. Wilson , The Ants (Springer, 1990).

[5] R. Hickling and R. L. Brown , “Analysis of Acoustic Communication by Ants,” Journal of the Acoustical Society of America 108, no. 4 (2000): 1920–1929, 10.1121/1.1290515. 11051518

[6] L. P. Casacci , J. A. Thomas , M. Sala , et al., “Ant Pupae Employ Acoustics to Communicate Social Status in Their Colony's Hierarchy,” Current Biology 23, no. 4 (2013): 323–327, 10.1016/j.cub.2013.01.010. 23394832

[7] K. Schönrogge , F. Barbero , L. P. Casacci , J. Settele , and J. A. Thomas , “Acoustic Communication Within Ant Societies and Its Mimicry by Mutualistic and Socially Parasitic Myrmecophiles,” Animal Behaviour 134 (2017): 249–256, 10.1016/j.anbehav.2016.10.031.

[8] M. A. Travassos and N. E. Pierce , “Acoustics, Context and Function of Vibrational Signalling in a Lycaenid Butterfly–Ant Mutualism,” Animal Behaviour 60, no. 1 (2000): 13–26, 10.1006/anbe.1999.1364. 10924199

[9] F. Barbero , J. A. Thomas , S. Bonelli , E. Balletto , and K. Schönrogge , “Queen Ants Make Distinctive Sounds That Are Mimicked by a Butterfly Social Parasite,” Science 323, no. 5915 (2009): 782–785, 10.1126/science.1163583. 19197065

[10] M. Sala , L. P. Casacci , E. Balletto , S. Bonelli , and F. Barbero , “Variation in Butterfly Larval Acoustics as a Strategy to Infiltrate and Exploit Host Ant Colony Resources,” PLoS ONE 9, no. 4 (2014): e94341, 10.1371/journal.pone.0094341. 24718496 PMC3981827

[11] L. P. Casacci , S. Bonelli , E. Balletto , and F. Barbero , “Multimodal Signaling in Myrmecophilous Butterflies,” Frontiers in Ecology and Evolution 7 (2019): 454, 10.3389/fevo.2019.00454.

[12] L. P. Casacci , F. Barbero , P. Ślipiński , and M. Witek , “The Inquiline Ant *Myrmica karavajevi* Uses both Chemical and Vibroacoustic Deception Mechanisms to Integrate Into Its Host Colonies,” Biology 10, no. 7 (2021): 654, 10.3390/biology10070654. 34356510 PMC8301377

[13] K. Fiedler , “European and North West African Lycaenidae (Lepidoptera) and Their Associations With Ants,” Journal of Research on the Lepidoptera 28, no. 4 (1991): 239–257, 10.5962/p.332216.

[14] K. Fiedler , “The Ant Associates of Lycaenidae Butterfly Caterpillars—Revisited,” Nota Lepidopterologica 44 (2021): 159–174, 10.3897/nl.44.68993.

[15] N. E. Pierce , M. F. Braby , A. Heath , et al., “The Ecology and Evolution of Ant Association in the Lycaenidae (Lepidoptera),” Annual Review of Entomology 47 (2002): 733–771, 10.1146/annurev.ento.47.091201.145257. 11729090

[16] N. E. Pierce and E. Dankowicz , “Behavioral, Ecological and Evolutionary Mechanisms Underlying Caterpillar‐Ant Symbioses,” Current Opinion in Insect Science 52 (2022): 100898, 10.1016/j.cois.2022.100898. 35257969

[17] P. J. DeVries , “Enhancement of Symbioses Between Butterfly Caterpillars and Ants by Vibrational Communication,” Science 248, no. 4959 (1990): 1104–1106, 10.1126/science.248.4959.1104. 17733373

[18] F. Riva , F. Barbero , S. Bonelli , E. Balletto , and L. P. Casacci , “The Acoustic Repertoire of Lycaenid Butterfly Larvae,” Bioacoustics 26, no. 1 (2017): 77–90, 10.1080/09524622.2016.1197151.

[19] J. Slater and N. Kraus , “The Role of Rhythm in Perceiving Speech in Noise: A Comparison of Percussionists, Vocalists and Non‐Musicians,” Cognitive Processing 17, no. 1 (2016): 79–87, 10.1007/s10339-015-0740-7. 26445880 PMC5019948

[20] M. Hartbauer and H. Römer , “Rhythm Generation and Rhythm Perception in Insects: The Evolution of Synchronous Choruses,” Frontiers in Neuroscience 10 (2016): 223, 10.3389/fnins.2016.00223. 27303257 PMC4885851

[21] C. De Gregorio , P. Antonini , E. W. Heymann , and M. Gamba , “Isochrony in Titi Monkeys Duets: Social Context as a Proximate Cause of Duets' Rhythm and Regularity,” Proceedings of the Royal Society B: Biological Sciences 292, no. 2041 (2025): 20242805, 10.1098/rspb.2024.2805. PMC11836696 39968619

[22] C. De Gregorio , M. Maiolini , T. Raimondi , et al., “Isochrony as Ancestral Condition to Call and Song in a Primate,” Annals of the New York Academy of Sciences 1537, no. 1 (2024): 41–50, 10.1111/nyas.15151. 38925552

[23] T. C. Roeske , O. Tchernichovski , D. Poeppel , and N. Jacoby , “Categorical Rhythms Are Shared Between Songbirds and Humans,” Current Biology: CB 30, no. 18 (2020): 3544–3555.e6, 10.1016/j.cub.2020.06.072. 32707062 PMC7511425

[24] C. De Gregorio , T. Raimondi , V. Bevilacqua , et al., “Isochronous Singing in 3 Crested Gibbon Species (*Nomascus* spp.),” Current Zoology 70, no. 3 (2024): 291–297, 10.1093/cz/zoad029. 39035758 PMC11255994

[25] C. De Gregorio , M. Gamba , and A. R. Lameira , “Third‐Order Self‐Embedded Vocal Motifs in Wild Orangutans, and the Selective Evolution of Recursion,” Annals of the New York Academy of Sciences 1549, 1 (2025): 219–229, 10.1111/nyas.15373. 40376956 PMC12309442

[26] W. Forstmeier and H. Schielzeth , “Cryptic Multiple Hypotheses Testing in Linear Models: Overestimated Effect Sizes and the Winner's Curse,” Behavioral Ecology and Sociobiology 65, no. 1 (2011): 47–55, 10.1007/s00265-010-1038-5. 21297852 PMC3015194

[27] A. J. Dobson . An Introduction to Generalized Linear Models, 2nd ed. (Chapman and Hall/CRC, 2001), 10.1201/9781420057683.

[28] D. Bates , M. Mächler , B. Bolker , and S. Walker , “Fitting Linear Mixed‐Effects Models Using lme4,” Journal of Statistical Software 67 (2015): 1–48, 10.18637/jss.v067.i01.

[29] R. V. Lenth , B. Banfai , B. Bolker , et al. (2024). emmeans: Estimated Marginal Means, aka Least‐Squares Means (Version 1.10.5) [Computer software], https://cran.r‐project.org/web/packages/emmeans/index.html.

[30] M. E. Brooks , K. Kristensen , K. J. V. Benthem , et al., “glmmTMB Balances Speed and Flexibility Among Packages for Zero‐Inflated Generalized Linear Mixed Modeling,” R Journal 9, no. 2 (2017): 378–400.

[31] R. S. Ferreira , C. Poteaux , J. H. C. Delabie , D. Fresneau , and F. Rybak , “Stridulations Reveal Cryptic Speciation in Neotropical Sympatric Ants,” PLoS ONE 5, no. 12 (2010): e15363, 10.1371/journal.pone.0015363. 21203529 PMC3008743

[32] C. De Gregorio , D. Valente , T. Raimondi , et al., “Categorical Rhythms in a Singing Primate,” Current Biology 31, no. 20 (2021): R1379–R1380, 10.1016/j.cub.2021.09.032. 34699799

[33] B. Chievers , T. Jonsson , O. J. Cadena Castaneda , and F. Montealegre‐Z , “Ultrasonic Reverse Stridulation in the Spider‐Like Katydid Arachnoscelis (Orthoptera: Listrosceledinae),” Bioacoustics Journal 23, no. 1 (2014): 66–77.

[34] T. Robillard , F. Montealegre‐Z , L. Desutter‐Grandcolas , P. Grandcolas , and D. Robert , “Mechanisms of High‐Frequency Song Generation in Brachypterous Crickets and the Role of Ghost Frequencies,” Journal of Experimental Biology 216, no. 11 (2013): 2001–2011, 10.1242/jeb.083964. 23430987

[35] H. Ma , Z. Wang , P. Han , et al., “Small Apes Adjust Rhythms to Facilitate Song Coordination,” Current Biology 34, no. 5 (2024): 935–945.e3, 10.1016/j.cub.2023.12.071. 38266649

[36] C. De Gregorio and A. Lameira , “Twice Times Two: Dual Mechanism for Double Rhythmic Meter in Orangutans and the Evolution of Human Song,” Iscience 29, no. 1 (2026): 114273, 10.1016/j.isci.2025.114273. 41492465 PMC12765161

[37] F. Barbero , S. Bonelli , J. A. Thomas , E. Balletto , and K. Schönrogge , “Acoustical Mimicry in a Predatory Social Parasite of Ants,” Journal of Experimental Biology 212, no. pt. 24 (2009): 4084–4090, 10.1242/jeb.032912. 19946088

[38] F. Barbero , D. Patricelli , M. Witek , et al., “ *Myrmica* Ants and Their Butterfly Parasites With Special Focus on the Acoustic Communication,” Psyche: A Journal of Entomology 2012, no. 1 (2012): 725237, 10.1155/2012/725237.

[39] M. A. Elgar , D. R. Nash , and N. E. Pierce , “Eavesdropping on Cooperative Communication Within an Ant‐Butterfly Mutualism,” Science of Nature 103, no. 9 (2016): 84, 10.1007/s00114-016-1409-5. 27679457

[40] M. D. Greenfield . Signalers and Receivers: Mechanisms and Evolution of Arthropod Communication (Oxford University Press, 2002), 10.1093/oso/9780195134520.001.0001.

[41] H. Römer , “Environmental and Biological Constraints for the Evolution of Long‐Range Signalling and Hearing in Acoustic Insects,” Philosophical Transactions of the Royal Society of London Series B: Biological Sciences 340, no. 1292 (1993): 179–185, 10.1098/rstb.1993.0056.

[42] K. Fiedler and U. Maschwitz , “Functional Analysis of the Myrmecophilous Relationships Between Ants (Hymenoptera: Formicidae) and Lycaenids (Lepidoptera: Lycaenidae): II. Lycaenid Larvae as Trophobiotic Partners of Ants – A Quantitative Approach,” Oecologia 75, no. 2 (1988): 204–206, 10.1007/BF00378598. 28310835

[43] R. B. Cocroft and R. L. Rodríguez , “The Behavioral Ecology of Insect Vibrational Communication,” Bioscience 55, no. 4 (2005): 323–334, 10.1641/0006-3568(2005)055[0323:TBEOIV]2.0.CO;2.

[44] I. Castellanos and P. Barbosa , “Evaluation of Predation Risk by a Caterpillar Using Substrate‐Borne Vibrations,” Animal Behaviour 72, no. 2 (2006): 461–469, 10.1016/j.anbehav.2006.02.005.

[45] J. E. Yack , M. L. Smith , and P. J. Weatherhead , “Caterpillar Talk: Acoustically Mediated Territoriality in Larval Lepidoptera,” Proceedings of the National Academy of Sciences 98, no. 20 (2001): 11371–11375, 10.1073/pnas.191378898. PMC58736 11562462

[46] S. M. Matheson , L. M. Turchen , E. Mauduit , and J. E. Yack , “Buzzing Boundaries: Tiny Caterpillars Vibrate to Defend Leaf Tip territories,” Journal of Experimental Biology 228, no. 7 (2025): jeb249796, 10.1242/jeb.249796. 39927736 PMC11993261

