# Supplementary material for: Rhythmic Signaling of Ants and Butterflies With Varying Degrees of Myrmecophily
Source: Ann N Y Acad Sci. 2026 Feb 24;1556(1):e70223. doi: 10.1111/nyas.70223 (PMC12932961; doi:10.1111/nyas.70223)
Supplement: Supplementary file 1 — Supporting Information Tables: nyas70223‐sup‐0001‐Tables.docx [file NYAS-1556-0-s002.docx]

**Supplementary materials**

**TABLE S1**. Post hoc comparisons of the LMM testing for the effect of species identity on t_k_ durations.

| **contrast** | **estimate** | **SE** | **z.ratio** | **p.value** |
| --- | --- | --- | --- | --- |
| alcon - argiades | 0.0799 | 0.196 | 0.408 | 1.000 |
| alcon - argus | -0.5941 | 0.226 | -2.625 | 0.2355 |
| alcon - bellargus | -0.2974 | 0.196 | -1.521 | 0.9132 |
| alcon - corindon | -0.6487 | 0.334 | -1.944 | 0.6879 |
| alcon - dispar | 0.2683 | 0.258 | 1.040 | 0.9943 |
| alcon - icarus | -0.0154 | 0.196 | -0.079 | 1.000 |
| alcon - myrmica | 0.2901 | 0.168 | 1.724 | 0.8234 |
| alcon - orion | -0.6178 | 0.207 | -2.987 | 0.0974 |
| alcon - phleas | 0.1832 | 0.226 | 0.812 | 0.9993 |
| alcon - Tetramorium | 0.1377 | 0.166 | -0.829 | 0.9991 |
| argiades - argus | -0.6739 | 0.223 | -3.024 | 0.0882 |
| argiades - bellargus | -0.3773 | 0.192 | -1.969 | 0.6707 |
| argiades - corindon | -0.7286 | 0.331 | -2.199 | 0.5058 |
| argiades - dispar | 0.1884 | 0.255 | 0.739 | 0.9997 |
| argiades - icarus | -0.0953 | 0.192 | -0.497 | 1.000 |
| argiades - myrmica | 0.2103 | 0.164 | 1.285 | 0.9715 |
| **argiades - orion** | -0.6977 | 0.203 | -3.436 | 0.0250 |
| argiades - phleas | 0.1034 | 0.222 | 0.465 | 1.000 |
| argiades - Tetramorium | -0.2176 | 0.161 | -1.348 | 0.9602 |
| argus - bellargus | 0.2967 | 0.223 | 1.331 | 0.9636 |
| argus - corindon | -0.0546 | 0.350 | -0.156 | 1.000 |
| argus - dispar | 0.8624 | 0.279 | 3.088 | 0.0736 |
| argus - icarus | 0.5787 | 0.223 | 2.593 | 0.2520 |
| **argus - myrmica** | 0.8842 | 0.199 | 4.435 | 0.0005 |
| argus - orion | -0.0237 | 0.233 | -0.102 | 1.000 |
| *argus - phleas* | 0.7773 | 0.250 | 3.113 | 0.0685 |
| argus - Tetramorium | 0.4564 | 0.198 | 2.310 | 0.4267 |
| bellargus - corindon | -0.3513 | 0.331 | -1.060 | 0.9933 |
| bellargus - dispar | 0.5657 | 0.255 | 2.219 | 0.4914 |
| bellargus - icarus | 0.2820 | 0.192 | 1.470 | 0.9299 |
| **bellargus - myrmica** | 0.5875 | 0.164 | 3.590 | 0.0147 |
| bellargus - orion | -0.3204 | 0.203 | -1.578 | 0.8919 |
| bellargus - phleas | 0.4806 | 0.222 | 2.163 | 0.5315 |
| bellargus - Tetramorium | 0.1597 | 0.161 | 0.989 | 0.9962 |
| corindon - dispar | 0.9170 | 0.372 | 2.467 | 0.3239 |
| corindon - icarus | 0.6333 | 0.332 | 1.910 | 0.7109 |
| corindon - myrmica | 0.9388 | 0.316 | 2.970 | 0.1020 |
| corindon - orion | 0.0309 | 0.338 | 0.091 | 10.000 |
| corindon - phleas | 0.8319 | 0.350 | 2.377 | 0.3814 |
| corindon - Tetramorium | 0.5110 | 0.315 | 1.623 | 0.8730 |
| dispar - icarus | -0.2837 | 0.255 | -1.112 | 0.9903 |
| dispar - myrmica | 0.0218 | 0.235 | 0.093 | 1.000 |
| **dispar - orion** | -0.8861 | 0.264 | -3.360 | 0.0320 |
| dispar - phleas | -0.0851 | 0.279 | -0.305 | 10.000 |
| dispar - Tetramori | -0.4060 | 0.233 | -1.741 | 0.8140 |
| icarus - myrmica | 0.3056 | 0.164 | 1.863 | 0.7413 |
| icarus - orion | -0.6024 | 0.203 | -2.963 | 0.1042 |
| icarus - phleas | 0.1987 | 0.222 | 0.893 | 0.9984 |
| icarus - Tetramori | -0.1223 | 0.162 | -0.756 | 0.9996 |
| **myrmica - orion** | -0.9080 | 0.177 | -5.131 | <.0001 |
| myrmica - phleas | -0.1069 | 0.199 | -0.538 | 1.000 |
| **myrmica - Tetramorium** | -0.4279 | 0.127 | -3.370 | 0.0310 |
| **orion - phleas** | 0.8010 | 0.232 | 3.450 | 0.0238 |
| orion - Tetramorium | 0.4801 | 0.175 | 2.745 | 0.1798 |
| phleas - Tetramorium | -0.3209 | 0.197 | -1.631 | 0.8692 |

**Table S2.** Post hoc comparisons of the LMM testing for the effect of species identity on the durations of intervals between trains.

| **contrast** | **estimate** | **SE** | **df** | **t.ratio** | **p.value** |
| --- | --- | --- | --- | --- | --- |
| Tetramorium - myrmica | -1.35616 | 0.317 | 94.5 | -4.274 | **0.0022** |
| Tetramorium - alcon | -3.10727 | 0.378 | 77.2 | -8.212 | **<.0001** |
| Tetramorium - argiades | -0.88793 | 0.318 | 51.4 | -2.791 | 0.1907 |
| Tetramorium - argus | -0.56722 | 0.438 | 60.1 | -1.296 | 0.9661 |
| Tetramorium - bellargus | -2.23499 | 0.357 | 64.6 | -6.259 | **<.0001** |
| Tetramorium - corindon | -1.52812 | 0.814 | 118.6 | -1.877 | 0.7306 |
| Tetramorium - dispar | -0.89519 | 0.461 | 52.8 | -1.942 | 0.6876 |
| Tetramorium - icarus | -2.71303 | 0.379 | 70.6 | -7.15 | **<.0001** |
| Tetramorium - orion | 0.82852 | 0.339 | 49.1 | 2.444 | 0.3615 |
| Tetramorium - phleas | -1.39645 | 0.388 | 52.2 | -3.595 | **0.0270** |
| myrmica - alcon | -1.75111 | 0.425 | 97.6 | -4.117 | **0.0037** |
| myrmica - argiades | 0.46823 | 0.373 | 76.7 | 1.256 | 0.9732 |
| myrmica - argus | 0.78894 | 0.479 | 74.8 | 1.648 | 0.8563 |
| myrmica - bellargus | -0.87884 | 0.406 | 86.6 | -2.162 | 0.5373 |
| myrmica - corindon | -0.17196 | 0.837 | 123.5 | -0.205 | 1.0000 |
| myrmica - dispar | 0.46096 | 0.5 | 65.5 | 0.922 | 0.9975 |
| myrmica - icarus | -1.35688 | 0.426 | 90.7 | -3.183 | *0.0690* |
| myrmica - orion | 2.18468 | 0.391 | 71.3 | 5.592 | **<.0001** |
| myrmica - phleas | -0.04029 | 0.434 | 69.7 | -0.093 | 1.0000 |
| alcon - argiades | 2.21933 | 0.426 | 68.8 | 5.211 | **0.0001** |
| alcon - argus | 2.54004 | 0.521 | 69.9 | 4.872 | **0.0003** |
| alcon - bellargus | 0.87227 | 0.456 | 76.7 | 1.914 | 0.7063 |
| alcon - corindon | 1.57915 | 0.862 | 116.6 | 1.832 | 0.7585 |
| alcon - dispar | 2.21207 | 0.541 | 62.8 | 4.089 | **0.0055** |
| alcon - icarus | 0.39423 | 0.473 | 80.3 | 0.833 | 0.9989 |
| alcon - orion | 3.93579 | 0.442 | 65.6 | 8.91 | **<.0001** |
| alcon - phleas | 1.71081 | 0.481 | 65.2 | 3.559 | **0.0269** |
| argiades - argus | 0.32071 | 0.479 | 57.3 | 0.669 | 0.9998 |
| argiades - bellargus | -1.34706 | 0.407 | 59.5 | -3.309 | *0.0552* |
| argiades - corindon | -0.64018 | 0.837 | 112.1 | -0.765 | 0.9995 |
| argiades - dispar | -0.00726 | 0.501 | 51.7 | -0.015 | 1.0000 |
| argiades - icarus | -1.8251 | 0.427 | 64.3 | -4.275 | **0.0029** |
| argiades - orion | 1.71645 | 0.391 | 48.2 | 4.386 | **0.0028** |
| argiades - phleas | -0.50852 | 0.435 | 50.8 | -1.169 | 0.9831 |
| argus - bellargus | -1.66777 | 0.506 | 63.6 | -3.295 | *0.0560* |
| argus - corindon | -0.9609 | 0.889 | 106.5 | -1.08 | 0.9915 |
| argus - dispar | -0.32797 | 0.584 | 56.3 | -0.562 | 1.0000 |
| argus - icarus | -2.14581 | 0.522 | 66.7 | -4.11 | **0.0049** |
| argus - orion | 1.39574 | 0.494 | 55.7 | 2.828 | 0.1745 |
| argus - phleas | -0.82923 | 0.529 | 56.7 | -1.569 | 0.8886 |
| bellargus - corindon | 0.70688 | 0.853 | 113.8 | 0.829 | 0.9990 |
| bellargus - dispar | 1.3398 | 0.526 | 57.3 | 2.546 | 0.3012 |
| bellargus - icarus | -0.47804 | 0.457 | 72.1 | -1.047 | 0.9930 |
| bellargus - orion | 3.06352 | 0.424 | 57.1 | 7.232 | **<.0001** |
| bellargus - phleas | 0.83854 | 0.464 | 58.1 | 1.807 | 0.7713 |
| corindon - dispar | 0.63292 | 0.901 | 100.9 | 0.702 | 0.9998 |
| corindon - icarus | -1.18492 | 0.862 | 114.5 | -1.374 | 0.9522 |
| corindon - orion | 2.35664 | 0.845 | 109.5 | 2.788 | 0.1767 |
| corindon - phleas | 0.13166 | 0.866 | 106.5 | 0.152 | 1.0000 |
| dispar - icarus | -1.81784 | 0.542 | 60.2 | -3.355 | **0.0487** |
| dispar - orion | 1.72372 | 0.514 | 50.6 | 3.352 | *0.0523* |
| dispar - phleas | -0.50126 | 0.548 | 52 | -0.915 | 0.9975 |
| icarus - orion | 3.54155 | 0.443 | 61.6 | 8.001 | **<.0001** |
| icarus - phleas | 1.31658 | 0.482 | 61.8 | 2.734 | 0.2091 |
| orion - phleas | -2.22497 | 0.45 | 49.5 | -4.941 | **0.0004** |

**TABLE S3**.  Post hoc comparisons of the GLMM testing for the effect of the degree of myrmecophily on the interval between trains.

| **contrast** | **estimate** | **SE** | **df** | **t.ratio** | **p.value** |
| --- | --- | --- | --- | --- | --- |
| NO - Low | -0.449 | 0.641 | 53.5 | -0.701 | 0.9554 |
| NO - Medium | 0.394 | 0.637 | 53.1 | 0.619 | 0.9715 |
| NO - High | -0.906 | 0.678 | 56.6 | -1.336 | 0.6701 |
| NO - Ants | 0.709 | 0.577 | 53.2 | 1.229 | 0.7347 |
| Low - Medium | 0.843 | 0.535 | 56.9 | 1.575 | 0.5191 |
| Low - High | -0.457 | 0.583 | 61.5 | -0.784 | 0.9343 |
| Low - Ants | 1.158 | 0.461 | 58.5 | 2.509 | 0.1025 |
| Medium - High | -1.301 | 0.580 | 61.1 | -2.243 | 0.1781 |
| Medium - Ants | 0.314 | 0.457 | 57.8 | 0.688 | 0.9584 |
| **High - Ants** | **1.615** | **0.512** | **63.8** | **3.152** | **0.0202** |
