# Supplementary material for: Rhythmic Signaling of Ants and Butterflies With Varying Degrees of Myrmecophily
Source: Ann N Y Acad Sci. 2026 Feb 24;1556(1):e70223. doi: 10.1111/nyas.70223 (PMC12932961; doi:10.1111/nyas.70223)
Supplement: Supplementary file 2 — Supporting Information Figures: nyas70223‐sup‐0002‐Figures.docx [file NYAS-1556-0-s001.docx]

**
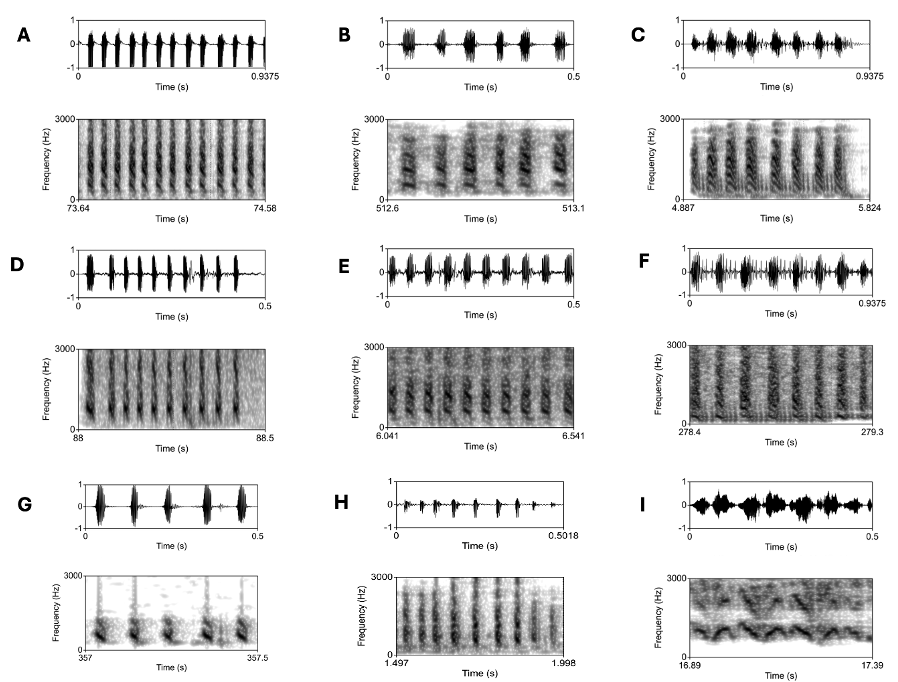
**

**Figure SM1.** Oscillograms (on top) and spectrograms (bottom) of the vibroacoustic signals emitted by the species investigated. A) *C. argiades*, B) *P. bellargus,* C) *P. argus,* D) *L. dispar,* E) *P. icarus,* F) *P. coridon*, G) *S. orion*, H) *L. phleas*, I) *Tetramorium. Myrmica* and *M. alcon* are shown in Figure 1.

**
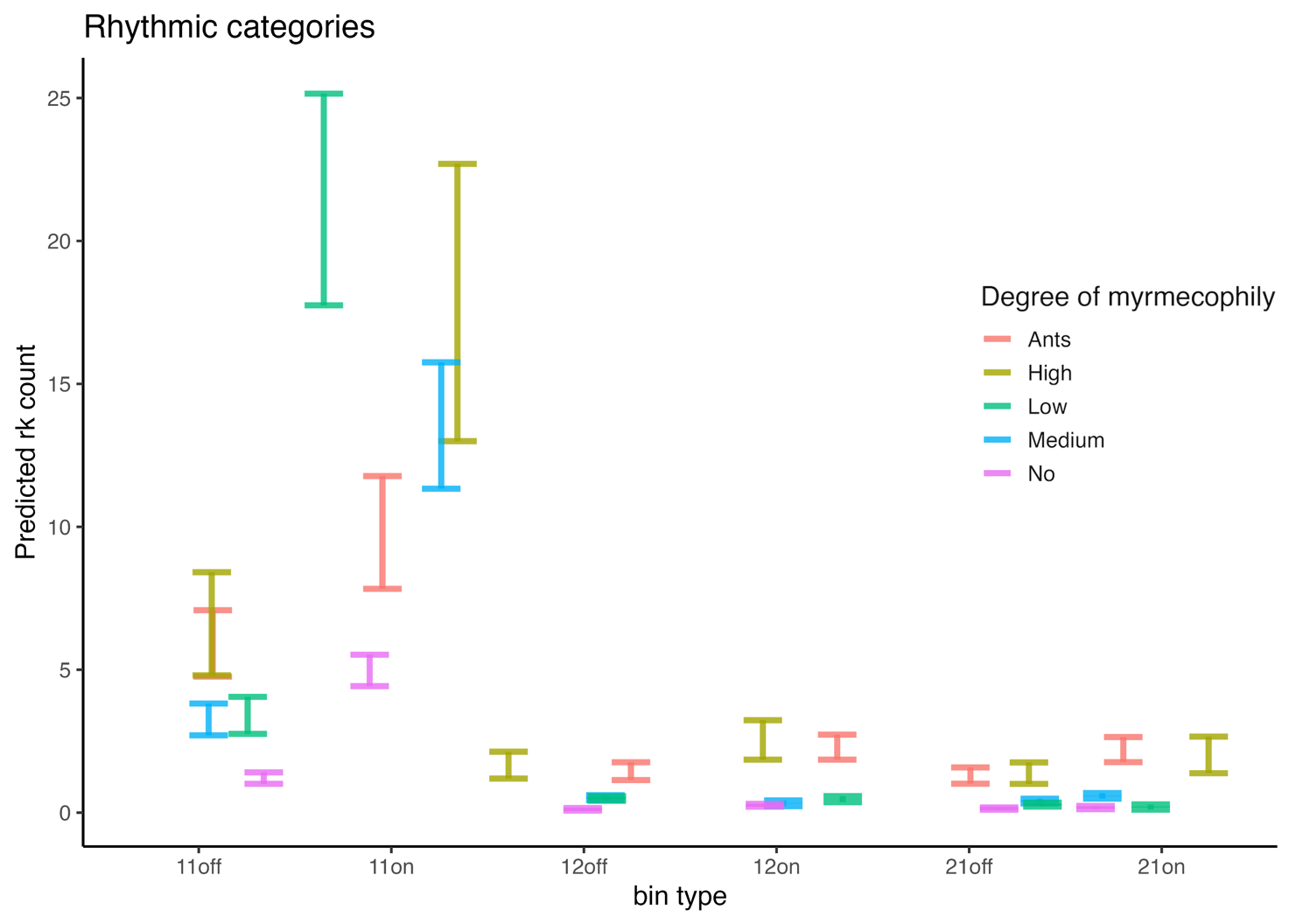
**

**Figure SM2**. Results of GLMM investigating ratio differences. 11off vs 11on was significant for all groups. 12 and 21 only for ants and species with a high degree of myrmecophily.
